# *N*,*N*′-Di-8-quinolyl-2,2′-(*o*-phenyl­ene­di­oxy)diacetamide

**DOI:** 10.1107/S1600536809041312

**Published:** 2009-10-23

**Authors:** Jing-Lin Wang

**Affiliations:** a Department of Biology and Chemistry, Changzhi University, Changzhi, Shanxi 046011, People’s Republic of China

## Abstract

In the title compound, C_28_H_22_N_4_O_4_, the mol­ecule lies on a crystallographic twofold axis. The quinoline ring is essentially planar (give max or rms deviation  0.0186 Å), and the dihedral angle between the quinoline ring and the central benzene ring is 19.1 (4)°. Intra­molecular N—H⋯(N,O) and C—H⋯O hydrogen bonds contribute to the formation of the roughly planar configuration. The crystal packing is stabilized by inter­molecular C—H⋯O hydrogen bonds, and weak π–π inter­actions between the pyridine rings and central benzene rings of the neighboring mol­ecules [centroid–centroid separation = 3.9009 (6) Å].

## Related literature

For background to the applications of amide-type acyclic polyethers, see: Guggi *et al.* (1977 ▶); Wen *et al.* (2002 ▶); West *et al.* (1992 ▶). For a related amide-type acyclic polyether structure, see: Wen *et al.* (2005 ▶).
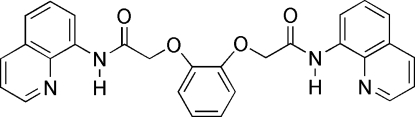


## Experimental

### 

#### Crystal data

C_28_H_22_N_4_O_4_
*M* *_r_* = 478.50
Orthorhombic, 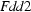

*a* = 32.648 (14) Å
*b* = 11.459 (4) Å
*c* = 12.516 (5) Å
*V* = 4682 (3) Å^3^
*Z* = 8
Mo *K*α radiation
μ = 0.09 mm^−1^
*T* = 294 K
0.20 × 0.14 × 0.12 mm

#### Data collection

Bruker SMART CCD area-detector diffractometer
Absorption correction: multi-scan (*SADABS*; Sheldrick, 1996 ▶) *T*_min_ = 0.982, *T*_max_ = 0.989
4687 measured reflections
1080 independent reflections
670 reflections with *I* > 2σ(*I*)
*R*_int_ = 0.060

#### Refinement

*R*[*F* ^2^ > 2σ(*F* ^2^)] = 0.037
*wR*(*F* ^2^) = 0.083
*S* = 1.00
1080 reflections
167 parameters
1 restraint
H atoms treated by a mixture of independent and constrained refinement
Δρ_max_ = 0.15 e Å^−3^
Δρ_min_ = −6.17 e Å^−3^

### 

Data collection: *SMART* (Bruker 2001 ▶); cell refinement: *SAINT* (Bruker 2001 ▶); data reduction: *SAINT*; program(s) used to solve structure: *SHELXTL* (Sheldrick, 2008 ▶); program(s) used to refine structure: *SHELXTL*; molecular graphics: *SHELXTL*; software used to prepare material for publication: *SHELXTL*.

## Supplementary Material

Crystal structure: contains datablocks I, global. DOI: 10.1107/S1600536809041312/bq2164sup1.cif

Structure factors: contains datablocks I. DOI: 10.1107/S1600536809041312/bq2164Isup2.hkl

Additional supplementary materials:  crystallographic information; 3D view; checkCIF report

## Figures and Tables

**Table 1:** Hydrogen-bond geometry (Å, °)

*D*—H⋯*A*	*D*—H	H⋯*A*	*D*⋯*A*	*D*—H⋯*A*
N2—H2*A*⋯O2	0.88 (4)	2.17 (4)	2.592 (5)	109 (3)
N2—H2*A*⋯N1	0.88 (4)	2.18 (4)	2.658 (5)	113 (3)
C7—H7⋯O1	0.93	2.32	2.915 (6)	121
C11—H11*B*⋯O1^i^	0.97	2.36	3.281 (6)	158

Symmetry code: (i) 
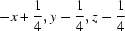
.

**Fig. 1.**
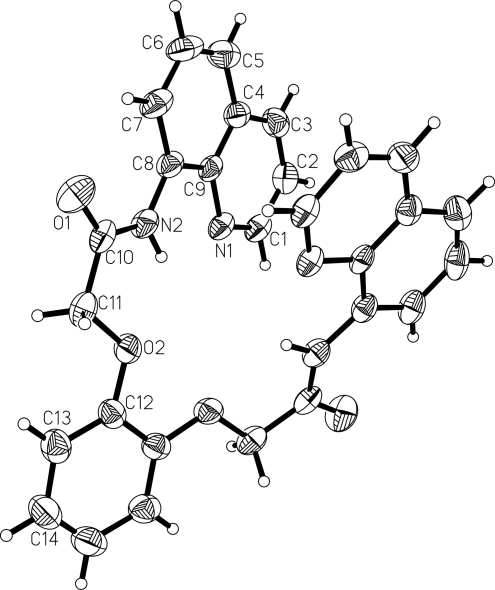
The molecular structure of (I), with atom labels and 20% probability displacement ellipsoids. [Symmetry code: (i) -x, 2-y, z].

**Fig. 2.**
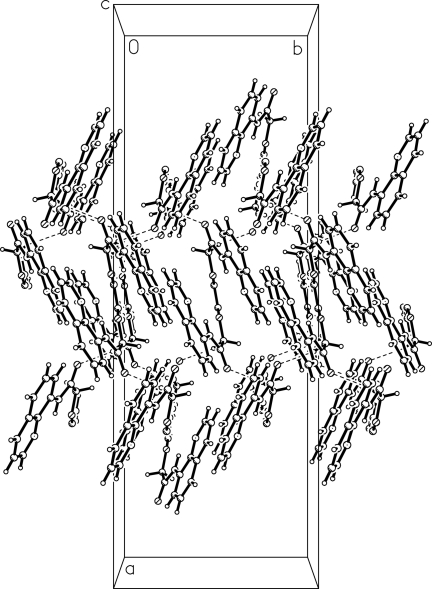
The packing diagram of (I) viewed down c-axis. Intermolecular H-bonds are indicated by dashed lines. H atoms were omitted for clearance.

## supplementary crystallographic information

### Comment

The amide-type acyclic polyethers have attracted wide attention in coordination chemistry and separation science because they have good complexing ability (West *et al.*, 1992) and high selectivity to metal ions (Wen *et al.*, 2002). In addition, this kind of compounds have been used successfully as active materials for ion-selective electrodes (Guggi *et al.*, 1977). Here, we report the synthesis and structure of the title compound.

The asymmetric unit of (I) contains one half-molecule, the other half being related by a crystallographic twofold axis (Fig. 1). All bond lengths and angles in (I) are within normal ranges, and comparable with those in the related compound (Wen *et al.*, 2005). The quinoline ring is essentially planar, with a dihedral angle of 2.1 (2)° between the benzene (C4—C9) ring and pyridine (C1—C4/C9/N1) ring. The dihedral angle between the quinoline ring and the central benzene ring is 19.1 (4)°. The amide N and C atoms are also planar configuration because the sum of the angles around atoms N2 and C10 are 359.3° and 360.0°, respectively. The intramolecular hydrogen bonds, N2—H2A···N1, N2—H2A···O2 and C7-H7···O1, form stable five- and six-membered rings, this being helpful to the formation of the planar configuration. The crystal packing is stabilized by intermolecular C11—H11B···O1 hydrogen bonds, and π-π interactions [short centroid-centroid separation = 3.9009 (6) Å] between the pyridine rings and central benzene rings of the neighboring molecules (Table 1 and Fig. 2).

### Experimental

Compound (I) was prepared according to the literature method of Wen *et al.* (2005). Yellow single crystals suitable for an X-ray diffraction study were obtained by slow evaporation of a petroleum ether-ethyl acetate solution (1:3 *v*/*v*) over a period of 10 d.

### Refinement

H atoms were positioned geometrically, with N—H = 0.86 Å and C—H = 0.95–0.99 Å, respectively, and constrained to ride on their parent atoms, with *U*_iso_(H) = 1.2 *U*_eq_(C,*N*). Absolute structure cannot be determined reliably.

### Crystal data

**Table:**

C_28_H_22_N_4_O_4_	*F*(000) = 2000
*M**_r_* = 478.50	*D*_x_ = 1.358 Mg m^−^^3^
Orthorhombic, *F**d**d*2	Mo *K*α radiation, λ = 0.71073 Å
Hall symbol: F 2 -2d	Cell parameters from 925 reflections
*a* = 32.648 (14) Å	θ = 2.5–20.7°
*b* = 11.459 (4) Å	µ = 0.09 mm^−^^1^
*c* = 12.516 (5) Å	*T* = 294 K
*V* = 4682 (3) Å^3^	Prism, yellow
*Z* = 8	0.20 × 0.14 × 0.12 mm

### Data collection

**Table:**

Bruker SMART CCD area-detector diffractometer	1080 independent reflections
Radiation source: fine-focus sealed tube	670 reflections with *I* > 2σ(*I*)
graphite	*R*_int_ = 0.060
phi and ω scans	θ_max_ = 25.0°, θ_min_ = 2.5°
Absorption correction: multi-scan (*SADABS*; Sheldrick,1996)	*h* = −38→34
*T*_min_ = 0.982, *T*_max_ = 0.989	*k* = −12→13
4687 measured reflections	*l* = −14→11

### Refinement

**Table:**

Refinement on *F*^2^	Primary atom site location: structure-invariant direct methods
Least-squares matrix: full	Secondary atom site location: difference Fourier map
*R*[*F*^2^ > 2σ(*F*^2^)] = 0.037	Hydrogen site location: inferred from neighbouring sites
*wR*(*F*^2^) = 0.083	H atoms treated by a mixture of independent and constrained refinement
*S* = 1.00	*w* = 1/[σ^2^(*F*_o_^2^) + (0.0372*P*)^2^] where *P* = (*F*_o_^2^ + 2*F*_c_^2^)/3
1080 reflections	(Δ/σ)_max_ < 0.001
167 parameters	Δρ_max_ = 0.15 e Å^−^^3^
1 restraint	Δρ_min_ = −0.16 e Å^−^^3^

### Special details

**Table:**

Geometry. All esds (except the esd in the dihedral angle between two l.s. planes) are estimated using the full covariance matrix. The cell esds are taken into account individually in the estimation of esds in distances, angles and torsion angles; correlations between esds in cell parameters are only used when they are defined by crystal symmetry. An approximate (isotropic) treatment of cell esds is used for estimating esds involving l.s. planes.
Refinement. Refinement of F^2^ against ALL reflections. The weighted R-factor wR and goodness of fit S are based on F^2^, conventional R-factors R are based on F, with F set to zero for negative F^2^. The threshold expression of F^2^ > 2sigma(F^2^) is used only for calculating R-factors(gt) etc. and is not relevant to the choice of reflections for refinement. R-factors based on F^2^ are statistically about twice as large as those based on F, and R- factors based on ALL data will be even larger.

### Fractional atomic coordinates and isotropic or equivalent isotropic displacement parameters (Å^2^)

**Table:**

	*x*	*y*	*z*	*U*_iso_*/*U*_eq_	
O1	0.13016 (8)	1.0567 (3)	0.5394 (3)	0.0891 (10)	
O2	0.03851 (7)	1.0165 (2)	0.39873 (19)	0.0631 (8)	
N1	0.00878 (10)	0.8564 (3)	0.6781 (2)	0.0567 (8)	
N2	0.07030 (10)	0.9673 (3)	0.5830 (3)	0.0588 (9)	
C1	−0.02253 (12)	0.8058 (3)	0.7257 (3)	0.0617 (11)	
H1	−0.0457	0.7900	0.6851	0.074*	
C2	−0.02300 (13)	0.7745 (4)	0.8338 (4)	0.0695 (12)	
H2	−0.0460	0.7392	0.8634	0.083*	
C3	0.01028 (14)	0.7961 (4)	0.8944 (4)	0.0712 (12)	
H3	0.0104	0.7760	0.9664	0.085*	
C4	0.04461 (13)	0.8488 (3)	0.8485 (3)	0.0588 (10)	
C5	0.08058 (15)	0.8758 (4)	0.9066 (4)	0.0772 (12)	
H5	0.0827	0.8552	0.9783	0.093*	
C6	0.11176 (16)	0.9313 (4)	0.8577 (4)	0.0818 (15)	
H6	0.1352	0.9484	0.8968	0.098*	
C7	0.11003 (13)	0.9644 (4)	0.7497 (4)	0.0717 (12)	
H7	0.1319	1.0034	0.7182	0.086*	
C8	0.07540 (11)	0.9384 (3)	0.6908 (3)	0.0562 (10)	
C9	0.04230 (11)	0.8797 (3)	0.7402 (3)	0.0513 (10)	
C10	0.09592 (12)	1.0223 (3)	0.5173 (3)	0.0599 (11)	
C11	0.08113 (11)	1.0392 (3)	0.4046 (4)	0.0635 (11)	
H11A	0.0866	1.1186	0.3818	0.076*	
H11B	0.0957	0.9867	0.3571	0.076*	
C12	0.02089 (10)	1.0103 (3)	0.2991 (3)	0.0583 (10)	
C13	0.04141 (13)	1.0219 (3)	0.2046 (3)	0.0728 (12)	
H13	0.0694	1.0369	0.2043	0.087*	
C14	0.02013 (13)	1.0111 (4)	0.1088 (4)	0.0905 (17)	
H14	0.0340	1.0194	0.0443	0.109*	
H2A	0.0455 (12)	0.947 (3)	0.560 (3)	0.077 (14)*	

### Atomic displacement parameters (Å^2^)

**Table:**

	*U*^11^	*U*^22^	*U*^33^	*U*^12^	*U*^13^	*U*^23^
O1	0.0467 (16)	0.098 (2)	0.122 (2)	−0.0231 (15)	−0.007 (2)	0.003 (2)
O2	0.0432 (15)	0.086 (2)	0.0598 (18)	−0.0090 (13)	0.0050 (14)	−0.0031 (14)
N1	0.0398 (18)	0.055 (2)	0.075 (2)	0.0006 (16)	−0.0048 (18)	−0.0033 (16)
N2	0.036 (2)	0.065 (2)	0.075 (3)	−0.0069 (18)	−0.0018 (19)	−0.0029 (17)
C1	0.046 (3)	0.055 (2)	0.084 (3)	0.002 (2)	−0.004 (2)	−0.001 (2)
C2	0.068 (3)	0.061 (3)	0.080 (3)	0.002 (2)	0.012 (2)	−0.003 (2)
C3	0.087 (3)	0.062 (3)	0.064 (3)	0.011 (3)	0.002 (3)	−0.005 (2)
C4	0.060 (3)	0.049 (2)	0.067 (3)	0.009 (2)	−0.010 (2)	−0.0103 (19)
C5	0.090 (3)	0.068 (3)	0.073 (3)	0.010 (3)	−0.020 (3)	−0.006 (2)
C6	0.076 (4)	0.068 (3)	0.102 (4)	0.014 (3)	−0.045 (3)	−0.017 (3)
C7	0.053 (3)	0.066 (3)	0.097 (3)	0.000 (2)	−0.018 (2)	−0.008 (2)
C8	0.044 (3)	0.056 (2)	0.069 (3)	0.004 (2)	−0.010 (2)	−0.0128 (19)
C9	0.045 (2)	0.040 (2)	0.069 (3)	0.0099 (18)	−0.004 (2)	−0.0094 (19)
C10	0.040 (2)	0.053 (3)	0.087 (3)	0.001 (2)	0.005 (2)	−0.007 (2)
C11	0.043 (2)	0.060 (2)	0.088 (3)	−0.0094 (18)	0.011 (2)	0.000 (2)
C12	0.067 (2)	0.052 (2)	0.055 (2)	0.005 (2)	0.005 (2)	0.0020 (18)
C13	0.079 (3)	0.067 (3)	0.072 (3)	0.003 (2)	0.014 (3)	0.009 (2)
C14	0.124 (4)	0.087 (4)	0.061 (3)	0.024 (4)	0.013 (3)	0.005 (3)

### Geometric parameters (Å, °)

**Table:**

O1—C10	1.217 (4)	C5—C6	1.348 (6)
O2—C12	1.375 (4)	C5—H5	0.9300
O2—C11	1.417 (4)	C6—C7	1.406 (6)
N1—C1	1.317 (4)	C6—H6	0.9300
N1—C9	1.369 (4)	C7—C8	1.382 (5)
N2—C10	1.332 (5)	C7—H7	0.9300
N2—C8	1.399 (5)	C8—C9	1.415 (5)
N2—H2A	0.89 (4)	C10—C11	1.504 (5)
C1—C2	1.400 (5)	C11—H11A	0.9700
C1—H1	0.9300	C11—H11B	0.9700
C2—C3	1.348 (5)	C12—C13	1.366 (5)
C2—H2	0.9300	C12—C12^i^	1.384 (7)
C3—C4	1.397 (5)	C13—C14	1.391 (6)
C3—H3	0.9300	C13—H13	0.9300
C4—C9	1.403 (4)	C14—C14^i^	1.339 (9)
C4—C5	1.416 (5)	C14—H14	0.9300
			
C12—O2—C11	117.8 (3)	C6—C7—H7	120.4
C1—N1—C9	116.7 (3)	C7—C8—N2	124.1 (4)
C10—N2—C8	129.3 (4)	C7—C8—C9	119.6 (4)
C10—N2—H2A	120 (3)	N2—C8—C9	116.3 (3)
C8—N2—H2A	111 (3)	N1—C9—C4	122.9 (3)
N1—C1—C2	124.0 (4)	N1—C9—C8	117.1 (3)
N1—C1—H1	118.0	C4—C9—C8	120.1 (4)
C2—C1—H1	118.0	O1—C10—N2	126.1 (4)
C3—C2—C1	119.2 (4)	O1—C10—C11	117.8 (4)
C3—C2—H2	120.4	N2—C10—C11	116.0 (3)
C1—C2—H2	120.4	O2—C11—C10	109.9 (3)
C2—C3—C4	119.7 (4)	O2—C11—H11A	109.7
C2—C3—H3	120.2	C10—C11—H11A	109.7
C4—C3—H3	120.2	O2—C11—H11B	109.7
C3—C4—C9	117.6 (4)	C10—C11—H11B	109.7
C3—C4—C5	123.3 (4)	H11A—C11—H11B	108.2
C9—C4—C5	119.1 (4)	C13—C12—O2	125.1 (3)
C6—C5—C4	119.7 (4)	C13—C12—C12^i^	120.0 (2)
C6—C5—H5	120.1	O2—C12—C12^i^	114.91 (16)
C4—C5—H5	120.1	C12—C13—C14	119.5 (4)
C5—C6—C7	122.3 (4)	C12—C13—H13	120.2
C5—C6—H6	118.8	C14—C13—H13	120.2
C7—C6—H6	118.8	C14^i^—C14—C13	120.5 (2)
C8—C7—C6	119.2 (5)	C14^i^—C14—H14	119.8
C8—C7—H7	120.4	C13—C14—H14	119.8
			
C9—N1—C1—C2	−0.6 (6)	C3—C4—C9—C8	176.9 (4)
N1—C1—C2—C3	−0.2 (7)	C5—C4—C9—C8	−0.8 (5)
C1—C2—C3—C4	−0.3 (6)	C7—C8—C9—N1	179.8 (3)
C2—C3—C4—C9	1.5 (6)	N2—C8—C9—N1	0.4 (4)
C2—C3—C4—C5	179.2 (4)	C7—C8—C9—C4	0.3 (5)
C3—C4—C5—C6	−177.1 (4)	N2—C8—C9—C4	−179.1 (3)
C9—C4—C5—C6	0.6 (6)	C8—N2—C10—O1	1.7 (6)
C4—C5—C6—C7	0.1 (7)	C8—N2—C10—C11	179.5 (3)
C5—C6—C7—C8	−0.6 (7)	C12—O2—C11—C10	−170.3 (3)
C6—C7—C8—N2	179.8 (4)	O1—C10—C11—O2	−168.2 (3)
C6—C7—C8—C9	0.4 (6)	N2—C10—C11—O2	13.8 (4)
C10—N2—C8—C7	0.3 (6)	C11—O2—C12—C13	1.5 (5)
C10—N2—C8—C9	179.7 (3)	C11—O2—C12—C12^i^	−179.1 (4)
C1—N1—C9—C4	2.0 (5)	O2—C12—C13—C14	178.5 (3)
C1—N1—C9—C8	−177.4 (3)	C12^i^—C12—C13—C14	−0.9 (7)
C3—C4—C9—N1	−2.5 (5)	C12—C13—C14—C14^i^	−0.4 (8)
C5—C4—C9—N1	179.7 (3)		

Symmetry codes: (i) −*x*, −*y*+2, *z*.
